# Social Media and Emotional Burnout Regulation During the COVID-19 Pandemic: Multilevel Approach

**DOI:** 10.2196/27015

**Published:** 2021-03-16

**Authors:** Ruosi Shao, Zhen Shi, Di Zhang

**Affiliations:** 1 The Donald P Bellisairo College of Communications The Pennsylvania State University University Park, PA United States; 2 The Research Center for Journalism and Social Development Renmin University of China Beijing China

**Keywords:** COVID-19, pandemic, emotion regulation, emotional exhaustion, multilevel approach, well-being, emotion, mental health, social media, perspective, strategy, effective, modeling, buffer

## Abstract

**Background:**

In February 2020, the Chinese government imposed a complete lockdown of Wuhan and other cities in Hubei Province to contain a spike of COVID-19 cases. Although such measures are effective in preventing the spread of the virus, medical professionals strongly voiced a caveat concerning the pandemic emotional burnout at the individual level. Although the lockdown limited individuals’ interpersonal communication with people in their social networks, it is common that individuals turn to social media to seek and share health information, exchange social support, and express pandemic-generated feelings.

**Objective:**

Based on a holistic and multilevel perspective, this study examines how pandemic-related emotional exhaustion enacts intrapersonal, interpersonal, and hyperpersonal emotional regulation strategies, and then evaluates the effectiveness of these strategies, with a particular interest in understanding the role of hyperpersonal-level regulation or social media–based regulation.

**Methods:**

Using an online panel, this study sampled 538 Chinese internet users from Hubei Province, the epicenter of the COVID-19 outbreak in China. Survey data collection lasted for 12 days from February 7-18, 2020, two weeks after Hubei Province was placed under quarantine. The sample had an average age of 35 (SD 10.65, range 18-78) years, and a majority were married (n=369, 68.6%).

**Results:**

Using structural equation modeling, this study found that intrapersonal-level (B=0.22; β=.24; *P*<.001) and interpersonal-level (B=0.35; β=.49; *P*<.001) emotional regulation strategies were positively associated with individuals’ outcome reappraisal. In contrast with intrapersonal and interpersonal regulations, hyperpersonal (social media–based) regulation strategies, such as disclosing and retweeting negative emotions, were negatively related to the outcome reappraisal (B=–1.00; β=–.80; *P*<.001).

**Conclusions:**

Consistent with previous literature, intrapersonal-level regulation (eg, cognitive reappraisal, mindfulness, and self-kindness) and interpersonal-level supportive interaction may generate a buffering effect on emotional exhaustion and promote individuals’ reappraisal toward the stressful situation. However, hyperpersonal-level regulation may exacerbate the experienced negative emotions and impede reappraisal of the pandemic situation. It is speculated that retweeting content that contains pandemic-related stress and anxiety may cause a digital emotion contagion. Individuals who share other people’s negative emotional expressions on social media are likely to be affected by the negative affect contagion. More importantly, the possible benefits of intrapersonal and interpersonal emotion regulations may be counteracted by social media or hyperpersonal regulation. This suggests the necessity to conduct social media–based health communication interventions to mitigate the social media–wide negative affect contagion if lockdown policies related to highly infectious diseases are initiated.

## Introduction

### Background

Since the index case of COVID-19 was first documented in Wuhan on December 1, 2019 [1], the COVID-19 pandemic has swept across the world. On January 23, 2020, the Chinese government imposed a complete lockdown of Wuhan and other cities in Hubei Province, the epicenter of the COVID-19 outbreak in China, to contain the spike in new cases. In an effort to *flatten the curve*, this measure stipulated that only one person from each household was permitted to go outside the home for provisions once every 2 days, except for medical reasons or employment at food stores, pharmacies, or hospitals. Following the order of complete social distancing, local residents in Hubei Province had to drastically modify their lifestyles. This was not only in Hubei, and this can now be observed in other parts of the world such as London. Although such measures are effective in containing the spread of the virus at the societal level, medical professionals strongly voiced a caveat concerning the pandemic emotional burnout at the individual level [2]. The pandemic itself, as well as the drastic social distancing policies, generates unprecedented stressors including perceived severe threats to personal safety, intense fears, strong feelings of being out of control, exhaustion, and loneliness, thus exerting compounding effects on individuals mentally, emotionally, and physically [3,4].

Given the affective cost of coping with the pandemic, health authorities worldwide, including the US Centers for Disease Control and Prevention, have called for more attention to daily emotion regulation among individuals [5]. According to the literature on social psychology and interpersonal communication, traditional emotion regulation strategies include cognitive reappraisal, self-kindness regulation, and interaction with one’s support network. In addition to these traditional adjustment strategies, social media has become an important channel for disclosing and regulating negative emotions [6,7]. During the lockdown, individuals in isolation turn to social media to seek and share health information, exchange social support, and express pandemic-generated feelings [8]. Although previous studies suggest that social media use can promote psychological well-being through satisfying individual’s need for belonging, receiving informational and emotional support, and reducing stress and loneliness [9], empirical evidence equally reveals that, during the COVID-19 pandemic, social media use exacerbates the anxiety, stress, and depression symptoms people have already experienced [8,10]. The effectiveness of social media–based emotion regulation, along with its relationship with the traditional regulation strategies, calls for a careful and systematic examination.

Additionally, existing COVID-19–related studies on the association between social media use and mental health focus on social media use alone without considering other possible methods of emotion regulation [8,10]. However, the extant literature on emotion regulation and well-being suggests that, although regulation effectiveness differs across each strategy, there is no single best and cure-all solution [7]. Instead, researchers suggest that a combination of several strategies and a certain level of flexibility in choosing regulation strategies predict more desirable outcomes [7]. Thus, researchers have called for adopting a multilevel approach to understanding the emotion regulation process within and across levels, along with the dynamics between regulation strategies across levels [11]. Based on a holistic and multilevel perspective, this study first examined how pandemic-related emotional exhaustion enacts regulation behaviors at each level and then evaluated the effectiveness of these regulation strategies, with a particular interest in depicting the interconnectedness among intrapersonal, interpersonal, and hyperpersonal regulation processes. Therefore, this study can present a more comprehensive picture of emotional regulation strategies during the COVID-19 lockdown than other studies.

This study also has potential practical implications for psychological intervention during lockdowns. The research is based on a survey of respondents from China’s Hubei Province, which was collected in early February 2020. During that period, people including experts knew little about the new coronavirus, and Hubei Province implemented perhaps the most stringent but effective COVID-19 lockdown measures in the world. An analysis of an extreme case of scientific uncertainty and emotional exhaustion can offer valuable practical experiences for professionals to design their psychological intervention schemes for similar situations in the future.

### Literature Review

#### Emotional Exhaustion in the COVID-19 Pandemic

Emotional exhaustion has been highlighted as one of the most prominent stress reactions signifying a state of feeling emotionally drained and a depletion of affective resources [12-14]. Exhaustion concerns have been examined in medical, educational, and organizational contexts, where individuals cope with stress and uncertainty on a routine basis [15-17]. The conceptualization of emotional exhaustion addresses its nature of being intensely affective and energy depleting, and therefore directly influences individuals’ appraisal and coping with the stressors.

Epidemic studies suggest that infectious diseases characterized by long duration, extra complexity, ambiguity, and excessive demands on coping resources could result in persistent psychopathological consequences such as acute distress, anxiety, and emotional exhaustion [18]. The results from two cross-sectional studies conducted in China and Italy show that nurses and medical professionals participating in COVID-19 emergency-related work experienced moderate to severe emotional exhaustion [19,20]. In addition to medical professionals, the public is also impacted by acute prevalent stressors during the outbreaks of infectious diseases. For example, Chinese college students reported significant emotional distress and psychological burnout in response to the severe acute respiratory syndrome (SARS) epidemic in 2003 [21,22], and similar emotional distress (eg, anxiety, stress, fear, and depression symptoms) was observed in various countries during the recent COVID-19 pandemic [10,23,24].

When experiencing emotional exhaustion, individuals initiate regulation processes to manage stressor-generated feelings and cope with the situation [25]. Empirical evidence in the past two decades suggests using various regulation strategies such as mindfulness, cognitive restricting, avoidance, and support seeking to facilitate adaptive stress appraisal and predict decreased emotional exhaustion [15,22]. Moreover, the results demonstrate that emotion regulation strategies serve as a buffer against the negative impacts of anxiety, fear, or stress and promote better psychological adjustment to the situation [7].

#### Reappraisal of the Pandemic Situation Through Multilevel Emotion Regulation

The goal to influence one’s emotional trajectory activates the emotion regulation process [26]. According to the transactional theory of stress and coping, individuals constantly appraise external stimuli within their environment, among which those appraised as threatening, harmful, or challenging generate negative emotions, further initiating the regulation process [25]. In the regulation processes, individuals enact various strategies, deliberately or unconsciously, to change the current affective state.

According to the transactional theory, the regulation process involves “constantly changing cognitive and behavioral efforts to manage external and/or internal demands that are appraised as taxing or exceeding the resources of a person” [25]. Individuals can either make efforts to manage the stressor or regulate resultant emotions directly. Therefore, regulation is conceptualized as a dynamic and adaptive process that restores the equilibrium between individuals’ appraisal of the environment and one’s coping resources. The transactional theory suggests assessing the effectiveness of the regulation process through cognitive reappraisal, including a re-evaluation of the situation’s nature (eg, from stressful to benign) and individuals’ coping ability (eg, from inadequate to adequate) toward it [27].

#### Intrapersonal-Level Emotional Regulation

Research on emotion regulation has identified various regulation strategies that vary in terms of their primary impacts on the emotion generative process. Empirical evidence suggests that individuals differ systematically in their use of regulation strategies and the reported effectiveness [28]. In the process model of emotion regulation, Gross [29] proposes to use cognitive reappraisal as a common emotion regulation strategy. Cognitive reappraisal is an antecedent-focused strategy that occurs before the emotion responses have been entirely generated. By reconstructing the emotion-eliciting situation differently, reappraisal alters its emotional impact and the subsequent emotional trajectory. Empirical evidence has demonstrated the effectiveness of reappraisal on downregulating negative emotions, improving environmental mastery, and promoting psychological well-being [28,30].

In recent decades, mindfulness has received more attention in cognitive and behavioral therapy, and has been recommended as an alternative to reappraisal regulation. Mindfulness regulation requires purposefully and nonjudgmentally focusing on the present moment and therefore promotes awareness of one’s current state and an openness to accept it [31,32]. The results from a daily diary study reveal that mindfulness regulation predicts emotional well-being, including a lower-level negative affect and a higher-level positive affect [33].

In addition to mindfulness regulation, researchers in positive psychology also suggest the use of self-kindness as an alternative to regulate negative affect. Self-kindness regulation entails understanding, gentleness, and love toward oneself in times of struggle; it not only is teachable through weeklong interventions but also demonstrates the buffering effects on stress and self-criticism [34,35].

Reappraisal, mindfulness, and self-kindness, although occurring at different stages during the emotion-generative process and each with a unique locus of focus, happen all at the intrapersonal level within an individual’s cognitive processing. These three regulation strategies are selected to reflect the characteristics of some commonly adopted intrapersonal-level regulations, rather than to provide a comprehensive review of emotion regulation. By involving these three strategies, this study first re-evaluates the extent that intrapersonal-level emotion regulation is associated with emotional exhaustion and outcome reappraisal in the context of the COVID-19 pandemic. In particular, we formed the following hypotheses (H):

- H1a: Pandemic-related emotional exhaustion will be positively associated with intrapersonal-level emotion regulation.
- H2a: Intrapersonal-level regulation will be positively associated with outcome reappraisal of the pandemic situation.

#### Interpersonal-Level Emotion Regulation and Support Seeking

Support seeking is commonly viewed as an active regulation behavior to cope with stressful situations and emotional distress [36]. It differs from the intrapersonal-level regulation process in its fundamentally social nature. As the “first act” in the process of supportive communication, support seeking is defined as “an intentional communicative activity to elicit supportive actions from others” [37]. When facing health-related stress and anxiety, individuals turn to their friends and family for emotional support to regain coping efficacy toward the situation. A cross-sectional study demonstrated that individuals who engaged in active support-seeking behaviors reported less depressive and anxiety symptoms when coping with SARS-related stressors [22].

The way people seek support from others varies in terms of the directness of one’s communication. A direct seeking behavior involves explicitly asking for assistance and clearly discussing the problem or distress [38]. In contrast, indirect seeking is often less informative about one’s problem and support needs, and often expresses one’s desire through nonverbal cues such as sighing, fidgeting, and avoiding eye contact [38,39]. The choice of direct and indirect seeking strategies is influenced by various factors including help seekers’ characteristics (eg, gender or social skills), appraisal of the current problem (eg, perceived stigma, attribution of the cause, or ego-relevance), and the relational nature with the support provider [36]. Although differing in their effectiveness, both support-seeking strategies are commonly adopted in the interpersonal-level regulation process and have demonstrated effects on improving emotional distress [40]. Thus, we hypothesize the following:

- H1b: Pandemic-related emotional exhaustion will be positively associated with interpersonal-level emotion regulation.
- H2b: Interpersonal-level emotion regulation will be positively associated with outcome reappraisal of the pandemic situation.

#### Hyperpersonal-Level Emotion Regulation

The prevalence of social technologies has expanded and transformed how individuals share and regulate emotional experiences. Recent studies have witnessed an increase in social media use and pandemic-related online expression, particularly aiming to manage the stress, anxiety, and emotional burnout toward COVID-19 [10,23]. During lockdown and social distancing, individuals express emotional experiences and release pent-up feelings to their social network and beyond through posting on social media [8]. Empirical evidence has shown that individuals who experience a higher intensity of stress provide more frequent and intimate self-disclosure on social media [41]. Therefore, research on emotion regulation proposes adopting online self-disclosure as an alternative regulation strategy and expects it to increase sharers’ ability to comprehend and further vent the negative affect [42].

However, the social context of the computer-mediated communication environment fundamentally changes the nature and the psychological outcomes of emotional disclosure. Unlike writing down feelings in a private diary, self-disclosure on social media always involves the presence of the *imaginary audience* who may or may not participate in concrete conversations. Therefore, social media constructs a hyperpersonal communication environment where social context cues such as the appearance and facial expressions of the conversational partner are constantly unavailable, and individuals perceive the possibility of others “reading” and “interacting” with their posts through an overattribution of “the abstract others” being present in the mediated environment [43].

Under the observation of the *imaginary audience*, sharing negative emotions, either through posting one’s feelings or retweeting emotional content that reflects one’s current affective state, may be regarded as *inappropriate* and could result in psychological maladjustment. The feeling of “inappropriateness” to negative disclosure feelings comes from both the “positivity norm” of internet culture and individuals’ self-presentation desires [44,45]. In particular, negative sharing receives less feedback, reduces perceived connectedness in the online community, and generates concerns about the social cost of a negative self-presentation. In line with the theoretical proposition, the results from a daily diary study reveal that disclosing negative emotions on social media increases negative affect, rather than neutralizing it [6].

In addition to posting feelings online, social media provides an alternative way to disclose one’s affective state, through retweeting emotional content. Retweeting emotional content, as another type of emotional disclosure, differs from the posting behavior by creating a connection between two individuals, the one who writes the original tweet and the one who shares it. Through retweeting, emotional experiences can be transmitted from one person to another and become *contagious*. Given that the COVID-19 outbreak has generated impacts on a large scale, individuals are more susceptible to take on other people’s stress, anxiety, and emotional burnout on social media [10]. Considering the effects of social media self-disclosure and emotion contagion, we further hypothesize that hyperpersonal-level emotion regulation, such as sharing feelings or retweeting emotional content on social media, may generate maladaptive effects.

- H1c: Pandemic-related emotional exhaustion will be positively associated with hyperpersonal-level emotion regulation.
- H2c: Hyperpersonal-level emotion regulation will be negatively associated with outcome reappraisal of the pandemic situation.

#### A Multilevel Approach to Emotion Regulation

The process of emotion regulation involves using internal, relational, and societal resources to manage individuals’ affective state and, thereby, is fundamentally a multilevel construct. Individuals dynamically adjust regulation strategies across contexts and use emotion regulation within and across levels to maximally succeed in pursuing their own idiosyncratic goals. A growing understanding suggests matching regulation strategies to environmental circumstances and evaluating the effectiveness of emotion regulation holistically, rather than focusing on individual strategies [46].

**Figure 1:**
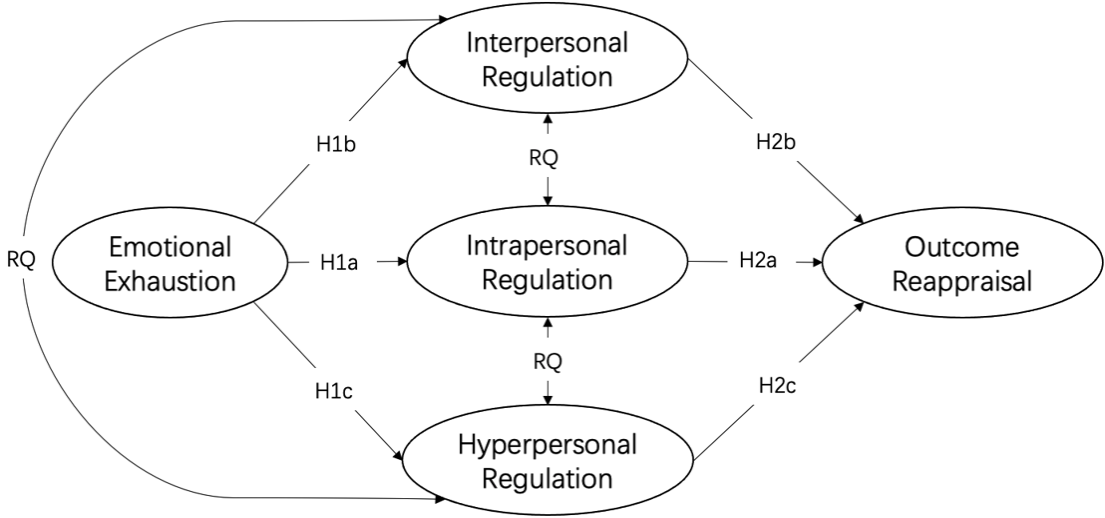
Conceptual model. H: hypothesis; RQ: research question.

In this study, we adopt a multilevel approach to depict the interconnectedness between regulation processes at intrapersonal, interpersonal, and hyperpersonal levels [11]. By incorporating a multilevel focus, we examine the system feature of emotion regulation and thereby reveal the mechanism of regulation effectiveness from a broader sense (see Figure 1 for the conceptual model). For example, when individuals engage in emotion regulation at one level, will they simultaneously use regulation strategies at other levels? Furthermore, will regulation processes at different levels generate cumulative effects or counteract each other on coping effectiveness? Thus, we came up with a research question (RQ) about the relationship between regulation processes at three levels.

- RQ: How are emotion regulation processes at intrapersonal, interpersonal, and hyperpersonal levels correlated with each other?

## Methods

### Sample and Procedure

An online survey was conducted to examine individuals’ emotional state, regulation strategies, and outcome reappraisal and coping during the COVID-19 outbreak. Data collection lasted for 12 days from February 7 to 18, 2020, two weeks after Hubei Province was placed under quarantine. During the survey period, participants were undergoing quarantine based on government policy and received a link to the online questionnaire.

The survey was first designed in English and then translated into Chinese by two bilingual communication researchers. The final version was further reviewed by the third bilingual researcher with expertise in public health to ensure the accuracy of the translation. Sociocultural background and linguistic nuances were recognized and reflected during the translation. Additionally, we checked the validity of the measurements originally developed in Western contexts. This study measured five latent variables including emotional exhaustion, intrapersonal-level emotion regulation, interpersonal-level emotion regulation, hyperpersonal-level emotion regulation, and outcome reappraisal. All these measurement scales were successfully applied to Chinese respondents in previous studies that demonstrated their validity in the Chinese context [47-51].

Participants (N=538; male: n=273; female: n=265) from China’s Hubei Province, where the COVID-19 pandemic first broke out, were recruited from an online survey service in China. After the outbreak in Hubei, the Chinese government implemented strict lockdown and social distancing policies, and most social interactions of people in Hubei took place on mediated channels, which gave the researchers a unique context to understand emotional regulation strategies. The study used quota sampling to draw participants, which is common for online panel research [52]. The quota sampling scheme used age group and gender as the criteria to design subgroups (there were roughly equal numbers of respondents in the six subgroups). The age groups included 18-29 years, 30-39 years, and 40 years or older. The final sample had an average age of 35 (SD 10.65; range 18-78) years and with a majority being married (n=369, 68.6%). The respondents reported very frequent use of social media during the lockdown: for WeChat and microblogs, the average score was 6.01 (SD 1.17) and, for online forums and WeChat Groups, the average scores was 5.47 (SD 1.40; both on a scale of 1-7).

Compared to Chinese census data [53], the sample recruited from the online panel was younger and had slightly more females, and the sample were exclusively internet users. Since the major goal of the study is to examine how emotional regulation through social media is associated with reappraisal, the online panel data can best satisfy our research agenda. The age and gender quota in the final sample is specified to test the proposed conceptual model. Considering that people younger than 35 years account for 82%-83% of China’s internet users [54], we oversampled this age group to reflect the majority of internet users in China.

### Measures

All measures were on a 7-point scale ranging from 1 (*not at all*) to 7 (*very much*) unless otherwise indicated.

#### Emotional Exhaustion

Emotional exhaustion was measured with six items adopted from the emotional exhaustion subscale in the Maslach Burnout Inventory–General Survey developed by Schaufeli and colleagues [55]. The Maslach Burnout Inventory is widely used in research to measure emotional exhaustion in various situations and has been validated in studies examining COVID-19–related burnout [19]. We removed three working-related items and kept the remaining six items to measure general emotional exhaustion toward COVID-19, since most people were not back at work when the data was collected. In particular, participants rated to what extent they feel emotionally drained and intense fear and anxiety during the lockdown (mean 20.25, SD 8.64; Cronbach α=.89).

#### Intrapersonal-Level Emotion Regulation

Three regulation strategies including cognitive reappraisal, self-kindness, and mindfulness were reported to assess intrapersonal-level emotion regulation. Considering that participants were experiencing pandemic-generated stress, we kept the questionnaire brief to increase the completion rate and reduce the response burden. Therefore, when measuring emotion regulation strategies, we only selected items with the highest factor loadings and that were suitable for the research context [56]. Given the one-dimensional nature of each regulation strategy, a short scale (even a single-item one) could demonstrate good validity against equivalent full-scale versions, if not be even preferable [57,58]. In this study, the results of the Cronbach alpha and Spearman–Brown formula, the latter of which was adopted for two-item measurements [59], demonstrated good reliabilities. *Cognitive reappraisal regulation* was measured with five items from the Emotion Regulation Questionnaire by Gross and John [28] asking the level that participants “change the way they think about the pandemic situation when they want to feel less negative emotion” (mean 25.94, SD 5.04; Cronbach α=.76). *Self-kindness regulation* was measured with five items based on the self-kindness subdimension in the Self-Compassion Scale by Neff [60]: “I give myself the caring and tenderness I need during the COVID-19 pandemic” (mean 24.86, SD 5.66; Cronbach α=.81). *Mindfulness regulation* was measured with three items adopted from the mindfulness subdimension in the Self-Compassion Scale by Neff [60]: “When I am feeling down during the pandemic, I try to approach my feelings with openness” (mean 15.54, SD 3.34; Cronbach α=.68).

#### Interpersonal-Level Emotion Regulation

Two types of support-seeking behaviors (direct and indirect seeking) were assessed based on measurements developed by Derlega and colleagues [36] to evaluate interpersonal-level emotion regulation. *Direct support seeking* was measured with four items (eg, “I tell people the exact emotions I am experiencing because of the pandemic during the supportive interaction” and “I ask people for help when feeling negative about the pandemic situation during supportive interactions”; mean 13.13, SD 6.42; Cronbach α=.89). *Indirect support seeking* was measured with four items (eg, “I sign a lot when talking about the pandemic situation during the supportive interaction”; mean 10.05, SD 5.66; Cronbach α=.85). In addition, *emotional expressivity in support-seeking conversation* was measured with two items from the Emotional Expressivity Scale developed by Kring and colleagues [61] (eg, “I express my feelings about the pandemic in my conversation”; mean 7.82, SD 3.18; *r*_Spearman-Brown_=0.77).

#### Hyperpersonal-Level Emotion Regulation

Regulation strategies on the hyperpersonal-level focus on two emotional expressive behaviors online, posting and retweeting. *Emotional expressive posting* was measured with two items from the Emotional Expressivity Scale developed by Kring and colleagues [61] (eg, “I express my feelings about the pandemic in my online post”; mean 7.24, SD 3.27; *r*_Spearman-Brown_=0.79). *Emotional expressive retweeting* was measured by asking respondents to what extent their retweeting content conveys “fear,” “anxiety,” and “stress” about the pandemic situation (mean 8.22, SD 4.53; Cronbach α=.85).

#### Outcome Reappraisal

Two types of outcome reappraisal were examined as dependent variables with measures from Holmstrom and Kim [62]. *Emotion-focused reappraisal* was measured with four items asking to what extent respondents, for example, “feel less stressful towards the pandemic situation.” *Problem-focused reappraisal* was measured with five items such as “I know more about how to cope with the pandemic situation.” An additive index of nine items was created (mean 47.88, SD 9.31; Cronbach α=.89).

## Results

### Analysis Strategy

Structural equation modeling was used to test the relationship between all components in the proposed model simultaneously while accounting for measurement error. We first ran a confirmatory factor analysis to assess the construct validity of the measurement model and then created a path model using the Lavaan package in R (R Foundation for Statistical Computing). We constructed and examined a five-factor model under maximum likelihood estimation, as the multivariate normality assumption was not violated. A correlation matrix of latent variables (composite indexes were computed) is shown in Table 1.

**Table 1:** Bivariate correlations between composite indexes of latent variables (N=538).

Variables		1	2	3	4		5		6	7	8	9
**1. Outcome reappraisal**												
	*r*	—^a^										
	*P* value	—										
**2. Emotional exhaustion**												
	*r*	–0.35	—									
	*P* value	<.001	—									
**3. Self-kindness**												
	*r*	0.16	0.15	—								
	*P* value	<.001	.001	—								
**4. Mindfulness**												
	*r*	0.15	0.12	0.67	—							
	*P* value	<.001	.005	<.001	—							
**5. Reappraisal**												
	*r*	0.32	0.03	0.61	0.58	—						
	*P* value	<.001	.47	<.001	<.001	—						
**6. Direct seeking**												
	*r*	–0.06	0.23	0.24	0.21		0.12		—			
	*P* value	.20	<.001	<.001	<.001		.007		—			
**7. Indirect seeking**												
	*r*	–0.15	0.25	0.15	0.10		0.01		0.71	—		
	*P* value	.001	<.001	<.001	.02		.84		<.001	—		
**8. Expressive talk**												
	*r*	0.00	0.18	0.12	0.11		0.10		0.44	0.35	—	
	*P* value	.94	<.001	.004	.009		.02		<.001	<.001	—	
**9. Expressive posting**												
	*r*	0.00	0.23	0.22	0.15		0.09		0.44	0.40	0.50	—
	*P* value	.94	<.001	<.001	.001		.03		<.001	<.001	<.001	—
**10. Expressive retweeting**												
	*r*	–0.31	0.52	0.05	0.02		–0.08		0.40	0.44	0.29	0.38
	*P* value	<.001	<.001	.23	.65		.07		<.001	<.001	<.001	<.001

^a^Not applicable.

### Measurement Model

In building the measurement model, the antecedent emotional exhaustion and outcome reappraisal were first-order latent variables. For the three levels of regulation strategies, three second-order latent variables were assessed. The second-order intrapersonal-level emotional regulation included cognitive reappraisal, self-kindness, and mindfulness; interpersonal-level regulation consisted of direct and indirect support seeking and emotional expressivity in support seeking conversation; and hyperpersonal-level regulation encompassed emotional expressive posting and emotional expressive retweeting. The measurement portion of the model demonstrated a good fit (χ^2^_841_/*df*=2.50; root mean square error of approximation [RMSEA]=0.053; standardized root mean square residual [SRMR]=0.067). A comparative fit index (CFI) was not computed to assess the model fit in this study since, according to statistician Kenny [63], a CFI is not informative when the RMSEA of the null model is less than 0.158 (in this case, it was 0.155).

### Path Model and Hypothesis Testing

**Figure 2:**
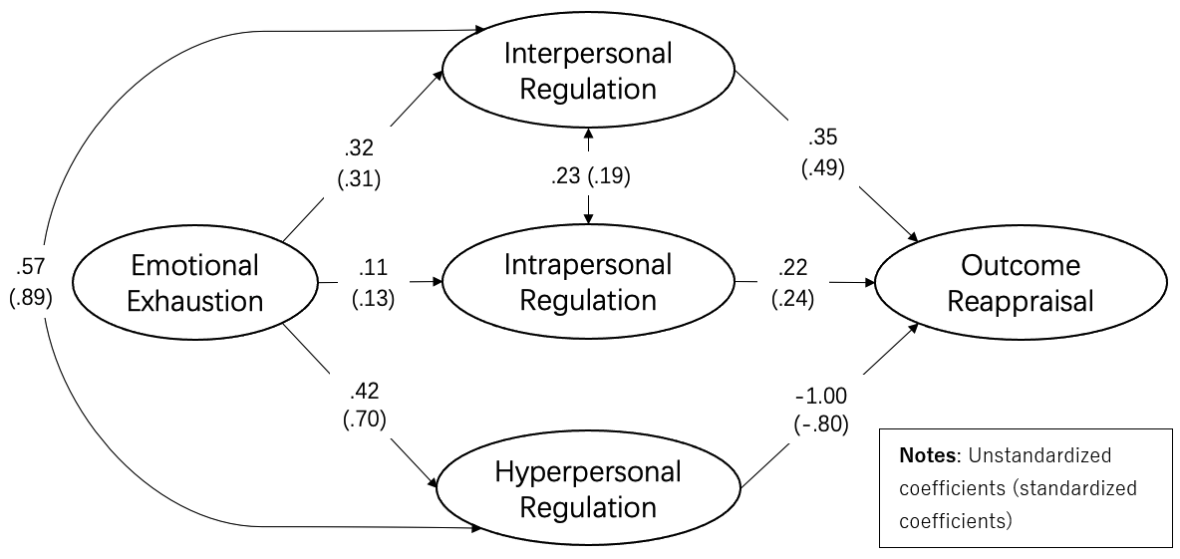
Revised model.

Further, we turned to the path model to examine the relationships among emotional exhaustion, three levels of emotion regulation, and outcome reappraisal. In the path model (Figure 2), emotional exhaustion was included as the exogenous variable and was left in the model to be associated with three levels of regulation strategies. Intrapersonal, interpersonal, and hyperpersonal emotion regulation were defined as three second-order latent factors in the model. Each of the three was allowed to be related to outcome reappraisal. To explore the relationships between three levels of the regulation process, we correlated the three regulation strategies (second-order latent variables in the middle of Figure 2). The hypothesized model demonstrated good fit (χ^2^_842_/*df*=2.68; RMSEA=0.056; SRMR=0.068). Figure 2 displays the path estimates that are statistically significant at the *P* level of .05. The following paragraphs show the results of the hypothesis testing.

H1a hypothesized a positive association between emotional exhaustion and intrapersonal-level emotion regulation. According to the results of the test, H1a was supported (B=0.11; β=.13; *P*=.01). H2a predicted that intrapersonal-level regulation is positively related to outcome reappraisal. The results suggest that H2a was supported (B=0.22; β=.24; *P*<.001).

H1b hypothesized that emotional exhaustion is positively associated with interpersonal-level emotion regulation. H1b was supported (B=0.32; β=.31; *P*<.001). H2b predicted that interpersonal-level emotion regulation has a positive association with outcome reappraisal. The results of the path model suggest that H2b was supported **(**B=0.35; β=.49; *P*<.001).

H1c predicted that emotional exhaustion is positively related to hyperpersonal-level emotion regulation, which was supported (B=0.42; β=.70; *P*<.001). H2c hypothesized a negative relationship between hyperpersonal-level emotion regulation and outcome reappraisal. The results were in line with our prediction (B=–1.00; β=–.80; *P*<.001).

The only research question in this study explores the relationships among the three levels of the regulation process. The results revealed that interpersonal-level emotional regulation is positively associated with intrapersonal-level regulation (B=0.23; β=.19; *P*=.001) and hyperpersonal-level regulation (B=0.57; β=.89; *P*<.001). In contrast, there exists no correlation between intrapersonal-level regulation and hyperpersonal-level regulation (B=0.003; β=.006; *P*=.94).

## Discussion

### Principal Findings

The COVID-19 pandemic has incurred unprecedented stressors, including the absence of vaccines, loneliness during social distancing, and concerns about the well-being of oneself and loved ones [2]. Given the evidence of affective cost among individuals across different cultures [2], it is crucial to understand and provide health interventions to individuals’ emotion regulation processes [8,23]. This study shows that residents in Hubei Province experienced a moderate level of emotional exhaustion during the COVID-19 outbreak and the ensuing unprecedented lockdown. The results find that individuals actively adopted cross-level regulation strategies to cope with the pandemic-generated burnout.

### Understanding and Intervening Emotion Regulation Systematically

As the epicenter of the COVID-19 outbreak, Hubei Province implemented stringent social distancing measures and mobility restrictions, such as residents-only entry policies in residential communities, compulsory wearing of face masks, and closing nonessential community services. To Hubei residents that had a lack of understanding toward the pathogenesis of the virus and the medical treatment and the vaccines that were under testing, the uncertainty and anxiety toward the pandemic accumulated to a high level. Under the unique circumstance, this study examined regulation processes among Hubei residents during the lockdown at intrapersonal, interpersonal, and hyperpersonal levels. Consistent with the previous studies [22,30-35], the results showed that intrapersonal-level regulation (eg, cognitive reappraisal, mindfulness, and self-kindness) and interpersonal-level supportive interaction seem to generate a buffering effect on emotional exhaustion and promote individuals’ reappraisal toward the stressful situation. However, according to the results of the study, hyperpersonal-level regulation exacerbates the experienced negative emotions and impedes reappraisal of the pandemic situation.

Based on a multilevel approach recommended by Cook and colleagues [11], this study examined three levels of emotion regulation processes simultaneously and depicted the interconnectedness between levels of regulation behaviors. The results reveal that the adoption of interpersonal-level regulation is commonly combined with intrapersonal or hyperpersonal regulation strategies. Individuals who either regulate one’s negative emotions online or internally also turn to friends and family for emotional support to cope with pandemic-generated affect. The complementary nature between interpersonal-level regulation and the other two levels points out the unique contribution of social interactions and the necessity of a certain degree of sociality in the regulation process.

In addition, the positive correlation between intra- and interpersonal level regulation behaviors raises concerns about the disparity regarding regulation resources and ability among individuals. In particular, individuals who are better at internal regulation (eg, cognitive reappraisal, self-kindness, and mindfulness) also engage more frequently in supportive interactions with one’s network, leading to a potential gap in emotional well-being between “skillful” regulator with rich support resources and the others who are bad at managing negative emotions and have fewer support resources. Thus, to eliminate the gap, community-wide interventions could prioritize those with fewer network resources and worse regulation ability to promote their emotional well-being.

### The Dark Side of Social Media–Based Emotion Regulation

In contrast with intrapersonal and interpersonal emotional regulations, social media–based regulation strategies, such as disclosing and retweeting negative emotions, generate maladaptive effects. The results in this study reveal that individuals who frequently disclose pandemic-related feelings and retweet COVID-19–related negative emotions on social media reported less reappraisal of the stressful situation. The possible maladaptive effects of social media use during the pandemic is consistent with several recent studies in the context of the current COVID-19 outbreak [8,10,64]. Based on the existing literature, this study analyzes social media in a more refined manner. Instead of measuring social media use as a whole, this study enhances our understanding by examining two specific active social media participation behaviors, namely, posting and retweeting. Through posting and retweeting emotional content, individuals use social media as a channel to disclose and regulate their negative emotions. However, the data of this study suggests that disclosing and sharing pandemic-related anxiety and fear on social media cannot help with relieving stress but may lead to a large-scale contagion of emotional burnout.

The possible counter-effects of hyperpersonal regulation may be due to the fact that the pandemic raises societal-level emotional burnout, during which many fellow social media users equally experience excessive demands on coping both emotionally and instrumentally [4]. This may limit their mental resources to provide supportive feedback to negative affective sharing on social media [4]. Therefore, disclosing negative feelings may cause others’ fear and anxiety, which further results in self-censorship of one’s disclosure behavior as “inappropriate” [44]. In addition, retweeting content that contains pandemic-related stress and anxiety may cause a digital emotion contagion [10]. Individuals who share other people’s negative emotional expressions on social media are likely to be affected by the negative affect contagion.

The results of the study sound stern alarms. This study finds that the size of the coefficients for emotional exhaustion’s association with intrapersonal regulation is the least, that for interpersonal regulation is mid-sized, and that for hyperpersonal regulation is the largest. In other words, in the era of social media, it is relatively hard for intrapersonal regulation to be activated, while emotional exhaustion can most easily kickoff hyperpersonal and interpersonal regulation. However, it should be noted that the outcomes of the latter two regulation strategies are completely opposite, and the size of the coefficient of hyperpersonal regulation is almost twice that of interpersonal regulation. This means that social media use (B=–1.00; β=–.80; *P*<.001) may counteract the benefits obtained through interpersonal **(**B=0.35; β=.49; *P*<.001) and intrapersonal (B=0.22; β=.24; *P*<.001) regulations. Without proper external interventions, individuals are unlikely to relieve their stress through easily obtained *tools* on their own during the lockdown in a pandemic.

### Practical Implications

The results in this study bear practical implications. The survey data was collected right after the outbreak of COVID-19 in Hubei Province, therefore providing a precious opportunity to examine individual’s mental well-being and emotion regulation behaviors during the initial stage of the public health risk. According to the crisis and emergency risk communication model, the initial stage of a public emergency is commonly characterized by a high level of uncertainty, a need for reducing stress, and a desire for reassuring self-efficacy [65]. Empirical evidence demonstrates that the high uncertainty and self-relevance nature of the pandemic’s initial stage generates moderate to high level emotional exhaustion among individuals. Further, the ability to actively and effectively regulate pandemic-generated emotional exhaustion varies significantly among individuals, and thus calls for public health communication interventions.

The social media–based emotion regulation process generates more impact on outcome reappraisal, compared to intrapersonal and interpersonal regulation. Considering the maladaptive effects of social media–based regulation, it is necessary to conduct effective health communication intervention in the emotional contagion on social media platforms if lockdown policies are to be initiated during public health crises. In the first step, public health agencies may consider using natural language processing techniques to conduct sentiment analysis of social media posts by individuals to closely monitor the stress level and anxiety tendency, and pinpoint the user segments with such symptoms. Further, these agencies may consider collaborating with social media platforms and using their content recommendation systems to share supportive messages with the target user segments to alleviate their stress and anxiety at the individual level, thus impeding the large-scale contagion of negative affect on social media.

Interpersonal-level regulation is most effective for managing pandemic-generated emotional exhaustion. Considering the benefits of social interactions on coping with pandemic-generated stress (interpersonal-level regulation), public health agencies may consider adopting emerging media technologies such as social robots and augmented and virtual reality to create opportunities for community residents to connect and talk with each other, which social distancing and lockdown policies have made difficult [66]. These new communication technologies could possibly create a mediated environment to fulfill individuals’ needs for social interaction and provide an effective channel for community-level intervention.

In addition, intrapersonal-level regulation, though demonstrated as effective and beneficial, can hardly be activated when experiencing emotional exhaustion. Public health interventions should consider focusing on improving awareness of individuals’ emotional state and providing guidance on conducting intrapersonal-level regulations. For example, health interventions could use mobile phone–based apps to help individuals practice mindfulness meditation and cognitive reappraisal. Public service advertisements could convey self-kindness messages to the public through broadcasting. Community-level regulation could hold online hangouts to help individuals engage in emotion regulation together virtually.

More importantly, this study implies the limitations of one-to-one psychological counseling. The results suggest that individuals in public health crises attempted various methods to regulate their negative affect. This means that, by incorporating the structural features of emotion regulation, health interventions may adopt a flexible combination of cross-level regulation strategies, based on the contexts in which individuals are embedded.

### Limitations and Future Research

The findings from this study should be considered alongside its limitations. A perennial problem with surveys is the bias of retrospective self-reports. Although emotion regulation is commonly defined as a goal-generated activity, some regulation processes may also happen subtly and unconsciously. In addition, the accuracy of recognizing, recalling, and reporting regulation processes varies among individuals based on their emotional intelligence level. Future studies could adopt a daily diary methodology to eliminate potential bias.

Another drawback of the study is the cross-sectional design, and readers should be cautioned about the causality. Future studies could adopt a longitudinal design and test the intervention effectiveness on a 2-week basis. In addition, this study examined individual’s emotion regulation behaviors at the initial stage of the COVID-19 outbreak. The initial stage of public health emergencies possesses a unique nature of uncertainty and a need for reassurance. Future studies could extend this study by focusing on other stages and making a systematic comparison.

By adopting a multilevel approach, this study examined emotion regulation processes that take place at different levels. The multilevel approach provides a systematic depiction of regulation processes while allowing for the flexibility of choosing and combining regulation strategies, therefore suiting the theoretical propositions of emotion regulation more appropriately. Future studies should consider adopting the multilevel approach when examining various regulation behaviors.

## Abbreviations

CFI: comparative fit index
H: hypothesis
MOE: Ministry of Education
RMSEA: root mean square error of approximation
RQ: research question
SARS: severe acute respiratory syndrome
SRMR: standardized root mean square residual
